# Feature-Rich Geometric and Electronic Properties of Carbon Nanoscrolls

**DOI:** 10.3390/nano11061372

**Published:** 2021-05-22

**Authors:** Shih-Yang Lin, Sheng-Lin Chang, Cheng-Ru Chiang, Wei-Bang Li, Hsin-Yi Liu, Ming-Fa Lin

**Affiliations:** 1 Department of Physics, National Chung Cheng University, Chiayi 621, Taiwan; sylin.1985@gmail.com; 2 Department of Electrophysics, National Chiao Tung University, Hsinchu 300, Taiwan; 3 Department of Physics, National Cheng Kung University, Tainan 701, Taiwan; davidgo86@livemail.tw (C.-R.C.); weibang1108@gmail.com (W.-B.L.); buttid41@gmail.com (H.-Y.L.)

**Keywords:** graphene, nanoscroll, first-principle

## Abstract

How to form carbon nanoscrolls with non-uniform curvatures is worthy of a detailed investigation. The first-principles method is suitable for studying the combined effects due to the finite-size confinement, the edge-dependent interactions, the interlayer atomic interactions, the mechanical strains, and the magnetic configurations. The complex mechanisms can induce unusual essential properties, e.g., the optimal structures, magnetism, band gaps and energy dispersions. To reach a stable spiral profile, the requirements on the critical nanoribbon width and overlapping length will be thoroughly explored by evaluating the width-dependent scrolling energies. A comparison of formation energy between armchair and zigzag nanoscrolls is useful in understanding the experimental characterizations. The spin-up and spin-down distributions near the zigzag edges are examined for their magnetic environments. This accounts for the conservation or destruction of spin degeneracy. The various curved surfaces on a relaxed nanoscroll will create complicated multi-orbital hybridizations so that the low-lying energy dispersions and energy gaps are expected to be very sensitive to ribbon width, especially for those of armchair systems. Finally, the planar, curved, folded, and scrolled graphene nanoribbons are compared with one another to illustrate the geometry-induced diversity.

## 1. Introduction

Condensed-matter systems purely made up of carbon atoms comprise diamond [1,2], few-layer graphenes [3,4,5], carbon nanotubes [6,7,8], graphene nanoribbons (GNR) [9,10,11,12], nanoscrolls [13,14,15,16] and C60-related fullerenes [17,18,19]. These systems exhibit very rich physical, chemical, and material properties, mainly owing to their special structural symmetries and varying dimensionality. Recently, one-dimensional carbon nanoscrolls (1D CNSs) have attracted much attention for their special geometric structure and electronic properties [13,14,17,18,20,21,22,23,24,25,26,27,28,29,30]. Each CNS can be regarded as a spirally-wrapped 2D graphene sheet with a 1D scroll structure. Unlike a carbon nanotube, which is a closed cylinder, a CNS is open at two edges. Clearly, CNSs possess flexible interlayer spaces to intercalate or to be susceptible to doping, indicating the high application potentials in hydrogen storage [24,25,31,32], lithium batteries [26,29,33,34], aluminum batteries [29], and mechanical devices [26,27]. However, regarding nanoscroll structures, the question remains whether they are perfectly spiral or not. The previous studies [35,36] on carbon nanotubes show that the non-cylindrical structures are more prone to exist in the large diameter cases due to the layer–layer interactions. Such effects are expected to play an important role in plastic CNSs. In this paper, we investigate the geometric and electronic properties of non-ideal CNSs, and these predicted results are innovative and interesting.

CNSs have been successfully produced by the different physical and chemical methods [13,14,17,18,21,22], including arc-discharge [17], high-energy ball milling of graphite [21]. and the chemical route. However, theoretical researches are rare and only focus on the ideal CNSs. Their electronic properties are predicted to be similar to those of the flat graphene nanoribbons, depending on edge structures and ribbon widths. In addition to the edge and quantum-confinement effects [37], the non-ideal CNSs are significantly affected by the curvature and stacking effects in terms of the structure stability and the electronic properties. In the past, many studies about curved ribbons [38,39,40,41] and few-layer graphene [42,43,44,45] have shown that the geometric structure is the key factor for the change of physical properties. The curved surface in CNSs leads to non-parallel $2p_{z}$ orbitals between the adjacent carbon atoms in the direction of bending, which results in hybridizations of carbon four orbitals [40,46,47]. However, the orbital hybridizations between two locally parallel surfaces are not found. Different stackings will have an impact on the layer–layer interactions and the free charge carriers [42,48,49]. These hybrid features in CNSs enable the possession of versatile and enhanced properties that are more adaptable in future electronic applications.

In this paper, the geometric and electronic properties of non-ideal carbon nanoscrolls are investigated by the first-principles calculations. This work is the first systematic study on two different kinds of CNSs with various widths and internal lengths. A new theoretical framework of charge distribution and the multi-orbital hybridization is implemented to explain the results. The dependences of formation energy and energy gaps on the internal length and the width are first obtained. A thorough discussion on electronic properties has not been published before. The essential properties, including the optimal geometric, formation energy, charge density, spin configuration, band structure, energy gap, and density of states, are determined by the completion and cooperation between the curvature and stacking effects. They possess basic properties similar to that of the flat nanoribbon, such as the zigzag systems being magnetic materials, three types of energy gaps classified in the armchair system, and the decreasing energy gaps resulting from the increased ribbon width. However, the hybrid structure accounts for the distinct properties. For instance, zigzag systems possess special electronic properties associated with the spin arrangements, the rule governing the size order of the energy gap is changed in the armchair system, and disregarding their system types, they all have smaller energy gaps compared with the flat nanoribbon. The predicted results could be verified by experimental measurements. These enriched electronic properties let the CNS have potential suitability not only in energy storage and machine components but also in electronic and spintronic devices.

## 2. Materials and Methods

The geometric and electronic structures of CNSs were studied by the Vienna ab initio simulation package [50,51] in the density-functional theory (DFT). The DFT-D2 method [52] was taken into account in order to describe the weak van der Waals interactions. The projector augmented wave method was utilized to characterize the electron–ion interactions. The exchange-correlation energy of the electron–electron interactions was evaluated within the local-density approximation. The wave functions were expanded by plane waves with the maximum kinetic energy limited to 500 eV. The k-point sampling is outlined by the Monkhorst-Pack scheme [53]. The 12×1×1 and 300×1×1 k-grids in the first Brillouin zone are, respectively, the settings used for the geometry optimization and band-structure calculations. The Hellmann–Feynman net force on each atom is smaller than 0.03 eV/Å. The axis of all nanoscrolls was set to be in the x-direction. In order to avoid interactions between the scrolled graphene superlattices of the adjacent unit cells, various vacuum spacings in the z-direction and y-direction are tested and a value of 15 Å is best for accurate and efficient calculations.

## 3. Results and Discussion

### 3.1. Geometric Properties and Formation Energy

**Figure 1:**
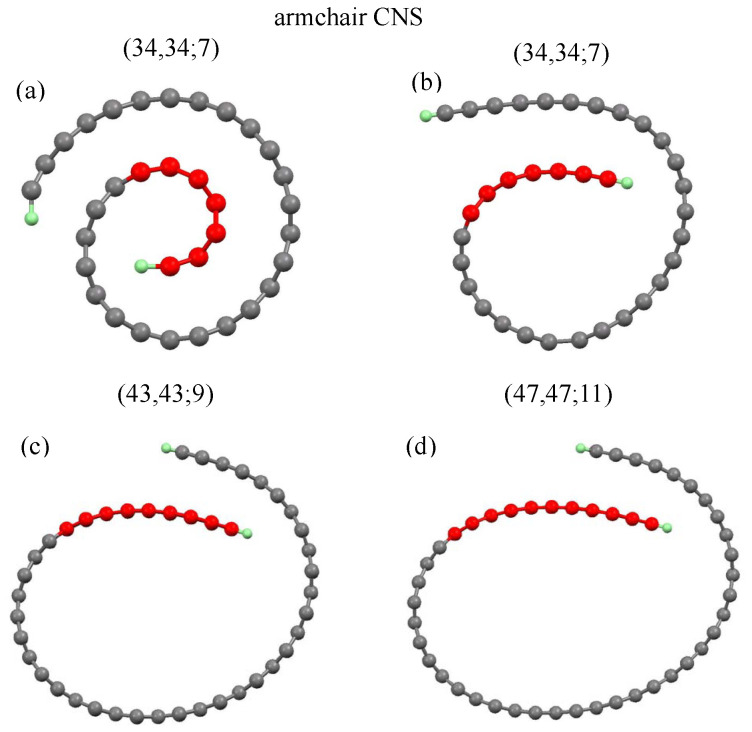
For armchair carbon nanoscrolls: the ideal structure of (**a**) (34,34;7) and the optimal structures of (**b**) (34,34;7), (**c**) (43,43;9); (**d**) (47,47;11).

**Figure 2:**
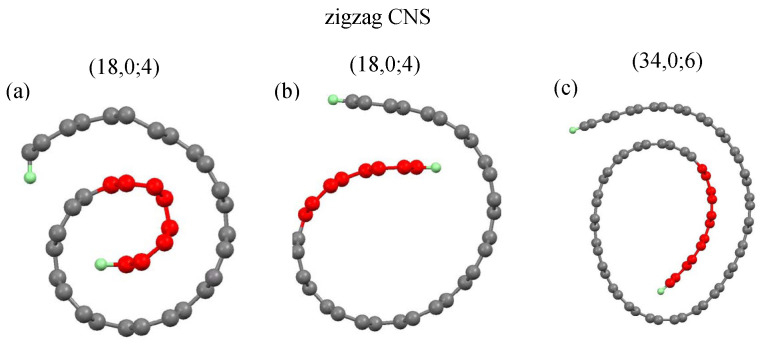
For zigzag carbon nanoscrolls: the ideal structure of (**a**) (18,0;4) and the optimal structures of (**b**) (18,0;4), (**c**) (34,0;6).

A CNS could be regarded as a rolled-up graphene sheet in the vector direction $R=ma_{1}+na_{2}$, or the (m,n) notation, where $a_{1}$ and $a_{2}$ are the basic vectors of a graphene sheet. Two open edges are saturated by hydrogen atoms (green color balls). Two typical longitudinal structures, armchair (m,m) and zigzag (m,0) CNSs were chosen for a model study, since they exhibit the unique geometric and electronic properties. Moreover, the optimal structures of CNSs are dependent on the initial conditions, including ribbon widths and internal lengths. The initial structures are kept at an arch shape, as shown in Figure 1a and Figure 2a.

The ribbon width (N${}_{y}$) is characterized by the number of dimer or zigzag lines along the transverse, and the internal length (N${}_{in}$) only counts the dimer or zigzag lines in the internal lengths (red balls). Armchair and zigzag CNSs, with their geometric characteristics, are defined by (N${}_{y}$,N${}_{y}$;N${}_{in}$) and (N${}_{y}$,0;N${}_{in}$), respectively.

**Figure 3:**
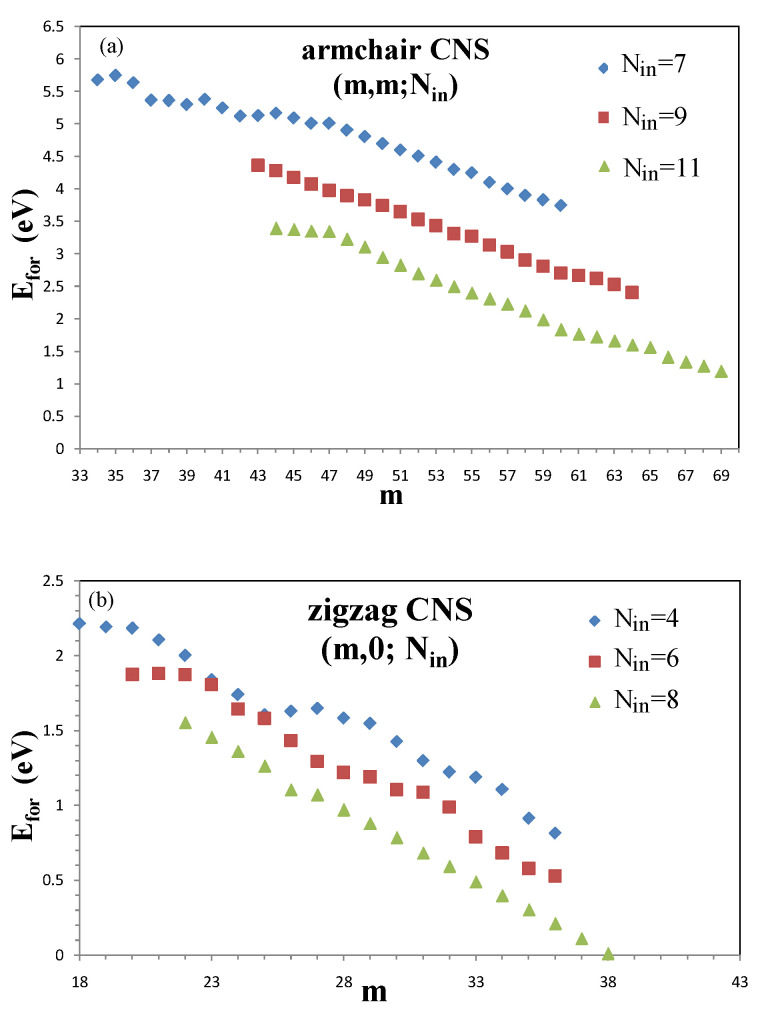
Formation energy of the scroll widths for (**a**) armchair CNS and (**b**) zigzag CNS with different internal lengths.

Before the self-consistent constraint is imposed, the initial arc structure is set to be an Archimedean spiral, as shown in Figure 1a and Figure 2a, and the carbon atoms on the curved surface are set to be the hexagonal structure. However, the relaxed optimal structure becomes less regular, as displayed in Figure 1b and Figure 2b. The internal length (N${}_{in}$) describes the ideal geometric structures before optimization, i.e., they only stand for initial conditions. The various initial conditions can result in different optimized geometric structures. We investigated three kinds of internal lengths for both armchair and zigzag CNSs with various scroll widths. The N${}_{in}$-dependent formation energy with various scroll widths was obtained. The results show that wider CNSs need to have a larger internal length as an initial condition to form the scroll shape, as shown by Figure 3a,b (discussed later). In other words, the increased initial N${}_{in}$ leads to different formation energy and critical formation width. However, the internal length in the optimized geometric structures can either increase or decrease. Interestingly, different initial N${}_{in}$ results in the same internal length in the optimized structure, as shown in Figure 1c,d. The (43,43;9) and (47,47;11) CNSs, respectively, have their N${}_{in}$ set to be 9 and 11, but their optimized structures exhibit the same internal length, i.e., the similar overlapping area. It should be noted that the red balls in Figure 1c,d are referred to their initial conditions.

The scroll geometry is sustained by the layer–layer interactions and simultaneously counterbalanced by strain forces. The reduced overlapping region caused by the insufficient width will hinder the formation of the scroll. The critical formation width of CNSs strongly depends on the internal length. Disregarding the periodic edge shape, all the interlayer distances are between 3.22 and 3.35 Å, which is close to the typical separation of graphene layers. A deeper understanding shows that all the interlayer configurations in CNSs are similar to those of the AB-layered carbon systems, owing to the higher cohesive energy presented in the AB stacking [6,7]. Perceivably, a CNS can be qualified as a stable structure, being determined by the sufficiently large width and overlapping length.

The formation of CNS is mainly dominated by two critical structure parameters: the internal length and the scroll width, as shown in Figure 3.

To hold the structure as a scroll, the required formation energy, defined as the energy difference between the total energy of a CNS and that of a flat GNR, is formulated as E${}_{for}$=E${}_{int}$ + E${}_{cur}$. E${}_{int}$ is the energy originating from the interlayer atomic interactions in the overlapping region. This term belongs to the binding force with a negative value. On the other hand, E${}_{cur}$ is the restoration force caused by the mechanic strain with a positive value. Given that E${}_{for}>0$, it is obvious that the strain energy is larger than the interlayer interactions. In searching for the minimum-width armchair systems, the critical width, which represents the smallest width to form a CNS with fixed N${}_{in}$, is found to be 34 associated with the (34,34;7) CNS, and its corresponding internal length is N${}_{in}=7$. As the width becomes larger, the corresponding increase of the overlapping region is responsible for pushing E${}_{int}$ negatively, and E${}_{cur}$ plunges due to its inverse proportionality to the square of the enlarged effective diameter [40]. Therefore, E${}_{for}$ decreases as the width increases, as shown in Figure 3a. Within the width range of m=34∼36, the interlayer distances are relatively large near the end of the overlapping region, leading to the weaker interlayer interactions and thus a smaller and smoother variation in the formation energy. As for m=37∼40, the stacking configurations in the overlapping region are close to a more stable AB stacking near the open end; therefore, the formation energy decreases more dramatically. As the width extends to the ranges m=41∼46 and m=47∼52, they all begin with a slow change but then evolve into a fast decrease in terms of the energy variation, i.e., the slope of the curve is gradually decreased in these two intervals. When the internal length grows, wider critical widths are obtained. N${}_{in}=9$ and 11 correspond to the critical widths m=43 and 47. To counter the decreased overlapping region, the wider critical width can reduce the mechanical strain and thus compensate for the loss of the interlayer interactions in forming the nanoscroll structure. In short, there are two factors taken into consideration in determining the formation energies of armchair nanoscrolls, the internal length and the scroll width. With the same internal length, the formation energy decreases for wider CNSs due to the reduction of mechanical strain. As for the nanoscrolls with the same width, a long internal length is energetically favored owing to a stronger interlayer interaction. These findings support the fact that a larger N${}_{in}$ results in a smaller E${}_{cur}$, as discussed previously.

**Figure 4:**
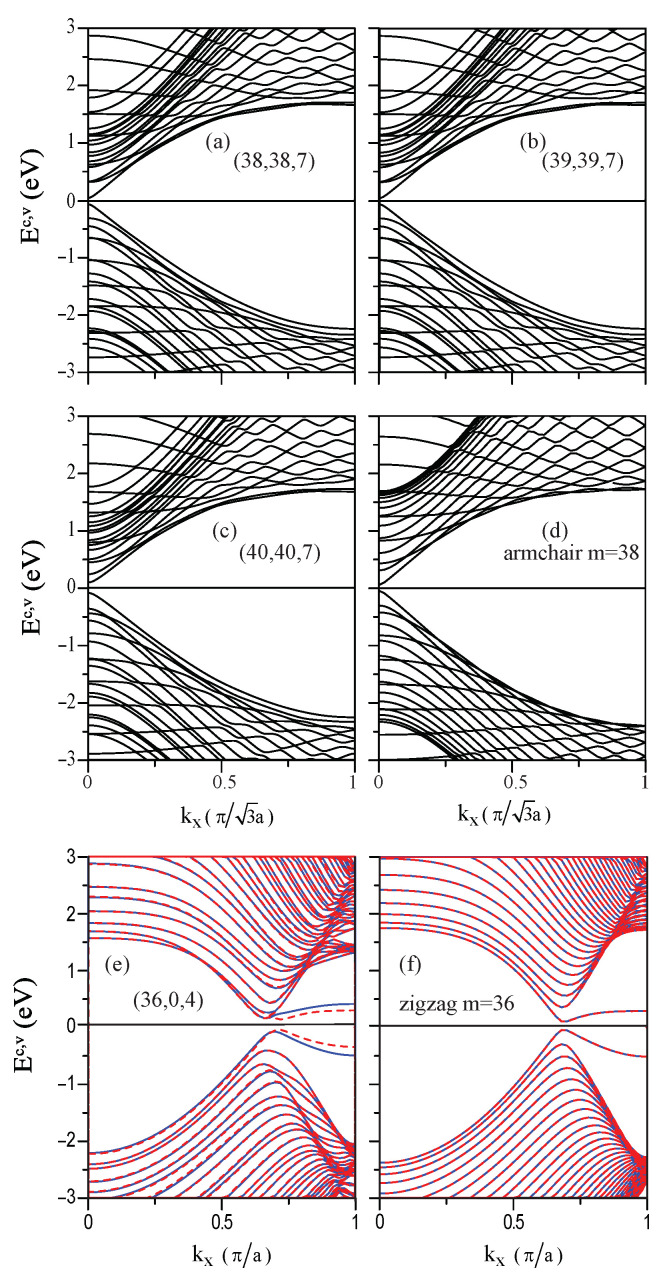
Band structures of the armchair system for (**a**) (38,38;7), (**b**) (39,39;7), (**c**) (40,40;7); (**d**) flat GNR with m = 38, and zigzag system for (**e**) (36,0;4); (**f**) flat GNR with m = 36.

The zigzag CNSs are similar to the armchair ones in the width dependence of formation energy. More specifically, when the minimum internal length is N${}_{in}=4$, the smallest zigzag CNS is (18,0;4). The scroll-width-dependent formation energy is shown in Figure 4b. Similar to what the armchair system has presented, we find a fluctuation in the dependence on scroll width, signifying that both systems share a common process during the geometric variation. N${}_{in}=6,8$ and 10 correspond to the critical widths m=20,22 and 23, respectively. With respect to the minimum-width system, the internal length is smaller in the armchair type than in the zigzag type, meaning that the former can overcome the larger restoration force caused by the mechanic strain. Therefore, the armchair CNSs are formed more easily and become more stable than those of the zigzag type.

### 3.2. Electronic Properties

The electronic properties of CNSs are deeply affected by edge shapes, widths, curvatures, and spin arrangements. An armchair (38,38;7) CNS, as shown in Figure 4a, exhibits a lot of 1D parabolic energy dispersions, in which the occupied valence bands are not symmetric to the unoccupied conduction bands about the Fermi level ($E_{F}=0$).

The energy bands are all doubly degenerate for both spin states: spin-up and spin-down. Each energy dispersion has the local minimum or maximum at $k_{x}=0$ and 1 and also at other wave vectors; that is to say, there are extra band-edge states except those at $k_{x}=0$ and 1. In the vicinity of $E_{F}$, the highest occupied state (HOS) and the lowest unoccupied state (LUS) occur at the same wave vector ($k_{x}=0.1$), which, thus, leads to a direct energy gap of $E_{g}=0.181$ eV, as shown in Table 1. Contrarily, the armchair (39,39;7) CNS (Figure 4b) possesses an indirect energy gap. Associated with this gap are the highest occupied and the lowest unoccupied states that appear, respectively, at $k_{x}=0.01$ and 0.13, and they are separated by a gap size of $E_{g}=0.112$ eV. These two energy bands are relatively smooth near $k_{x}$=0 without obvious dispersions. Such 3N-width characteristic is similar to that of the flat graphene nanoribbon [37]. Both the armchair (40,40;7) CNS with a (3N+1) width (Figure 4c) and the armchair (38,38;7) CNS with a (3N+2) width have direct energy gaps. The important differences between them are that the former has a smaller gap of $E_{g}=0.323$ eV and strongly non-monotonous energy dispersions. In addition, energy spacing of the $k_{x}=0$ state between two energy bands nearest to $E_{F}=0$ is higher than the energy gap. Apparently, there are certain important differences among the 3N-, (3N+1)- and (3N+2)-width systems. On the other hand, the CNSs are in sharp contrast to the flat GNRs (Figure 4d). The latter possesses a pair of monotonous parabolic energy dispersions nearest to $E_{F}=0$, in which their $k_{x}=0$ states determine a direct energy gap. Moreover, their energy gaps are a bit larger than those of the former, e.g., energy gaps of the m=38 systems.

**Figure 5:**
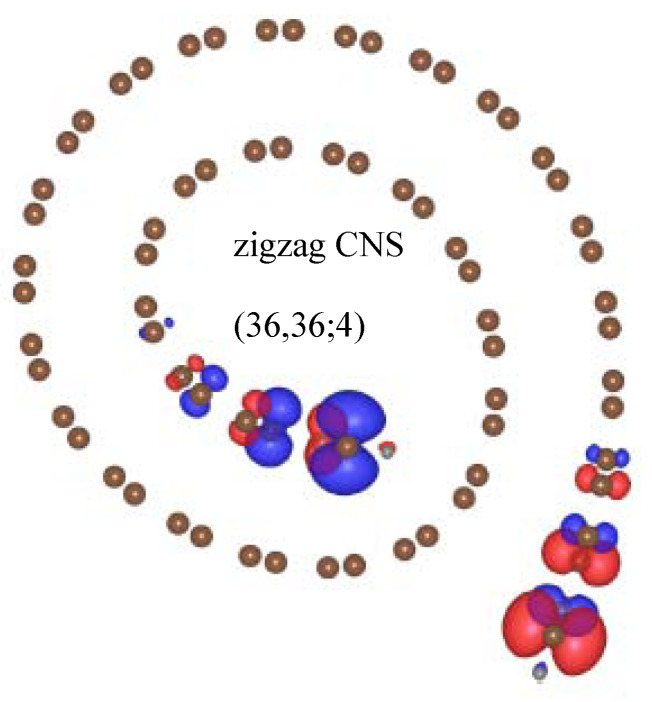
The charge distribution of spin-up and spin-down states, indicated by red and blue regions, respectively.

Electronic structures of zigzag CNSs are enriched by the anti-ferromagnetic spin configuration at two edges, as shown in Figure 4e for the (36,0;4) CNS. Most of the energy is doubly degenerate, while there exists the spin-up and spin-down splitting bands near $E_{F}=0$. This could be clearly understood from the spin-up and spin-down charge distributions in Figure 5 (red and blue colors) since for the distinct spin states, the H-passivated carbon atoms at each end of the open structure tend to interact differently with the surrounding atoms.

The four splitting bands have weak energy dispersions, in which they are mainly contributed to by the local edge atoms. Such bands will determine two kinds of spin-dependent energy gaps. The energy gaps belonging to the direct type appear approximately at $k_{x}=2/3$. Noticeably, the spin-up gap, 0.18 eV, is smaller than the spin-down gap, 0.23 eV. In comparison, for a flat GNR, four flat bands are partially degenerate and form two bands (Figure 4f), since the same end-structure environment in flat GNRs results in no difference for the edge effects from two ends. Again, the energy gap at $k_{x}=2/3$ is a direct one, with its size being at 0.14 eV.

### 3.3. Charge Distributions

**Figure 6:**
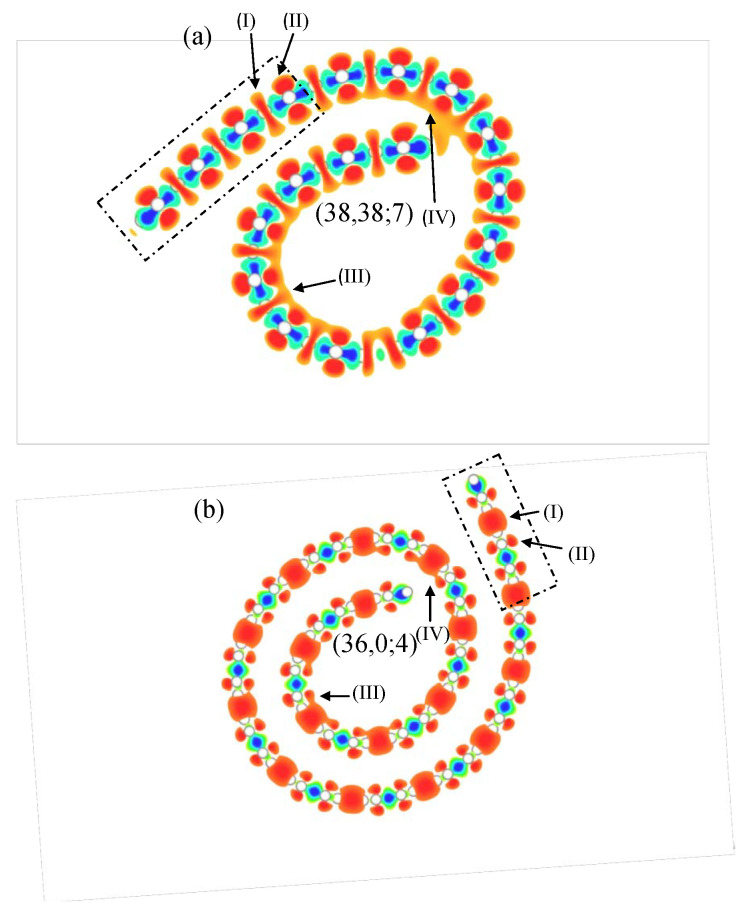
The charge distribution for (**a**) armchair (38,38;7) CNS; (**b**) zigzag (36,0;4) CNS. (II) are the 2 pz orbitals and (I) are the other three orbitals. (III) and (IV) are the serious orbital hybridizations.

The charge distribution on CNSs, which is very useful in understanding the hybridizations of orbitals (or the orbital bondings) and the low-lying energy bands, is significantly affected by the curved surface [47,54]. The variation of charge distribution created by subtracting the carrier density of an isolated carbon (a hollow circle) from that of a CNS is clearly illustrated in Figure 6a,b.

As for the planar region of the armchair (38,38;7) CNS, resembling a flat GNR as enclosed by the rectangle in Figure 6a, the 2s, $2p_{x}$, and $2p_{y}$ orbitals of one carbon atom interact with those of the nearby carbon atoms to form the σ bonds ((I) in orange shades). The charge densities are concentrated at the bond locations in the middle of the two binding atoms and significantly lowered for the remaining parts of the carbon atoms to create the depletion zones, as indicated by the blue shades. Moreover, the $2p_{z}$ orbitals ((II) in orange shades) perpendicular to the plane can interact with their nearest neighbors to form the π bonds. Induced by the curvature effect, there are two main causes that can contribute to the orbital hybridization. One is that the non-parallel $2p_{z}$ orbitals can lead to the σ bonds in addition to the π bonds. Another is the hybridization of four orbitals that is also responsible for introducing the complex π and σ bonds. Associated with these bond formations are the serious hybridizations that take place on the internal side of the curved surface, as shown in (III) and (IV). The direct impact from these hybridizations is reflected in the significant variation of the low-lying band structure, including the non-monotonic energy dispersions associated with the strongly hybridized atomic orbitals and the energy gap due to the $k_{x}\neq 0$ state (Figure 4a–c). The aforementioned changes to the band structure are in good agreement with the previous studies on the carbon nanotube and curved GNR. A further deduction from the curvature effect is that the dumbbell shape of $2p_{z}$ orbitals makes the charge distribution thicker on the nanoscroll surface towards the outside but thinner on the surface towards the inside. These larger $2p_{z}$ orbitals on the outer surface provide an ideal environment to bond with H, Li and other atoms, showing the possibilities in energy storage and electronic nano-devices.

The zigzag CNS (Figure 6b) and the armchair CNS partially share a similar charge distribution. In the planar region, they both have their π bonds created by the $2p_{z}$ orbitals (II) and their σ bonds formed from the 2s, $2p_{x}$, and $2p_{y}$ orbitals (I). Unlike the armchair configuration, the zigzag orbital hybridization in the curved regions (III and IV) appears to be weaker due to the longer distance between carbon atoms. As we move to the outer portion of the nanoscroll, the decreased curvature can reduce the hybridization to a trivial level. That is to say, the very weak hybridization is presented at the outer section. Speaking of the different features of energy bands in the zigzag CNS, the energy bands near $E_{F}$ are mainly contributed to by the carbon atoms at the two open edges. Given that spin-up and spin-down states are ignored, the distributions of the charge density at these two edges almost remain unchanged. Therefore, the energy bands in the zigzag case are not much different from those presented in the flat GNR.

### 3.4. Density of States

**Figure 7:**
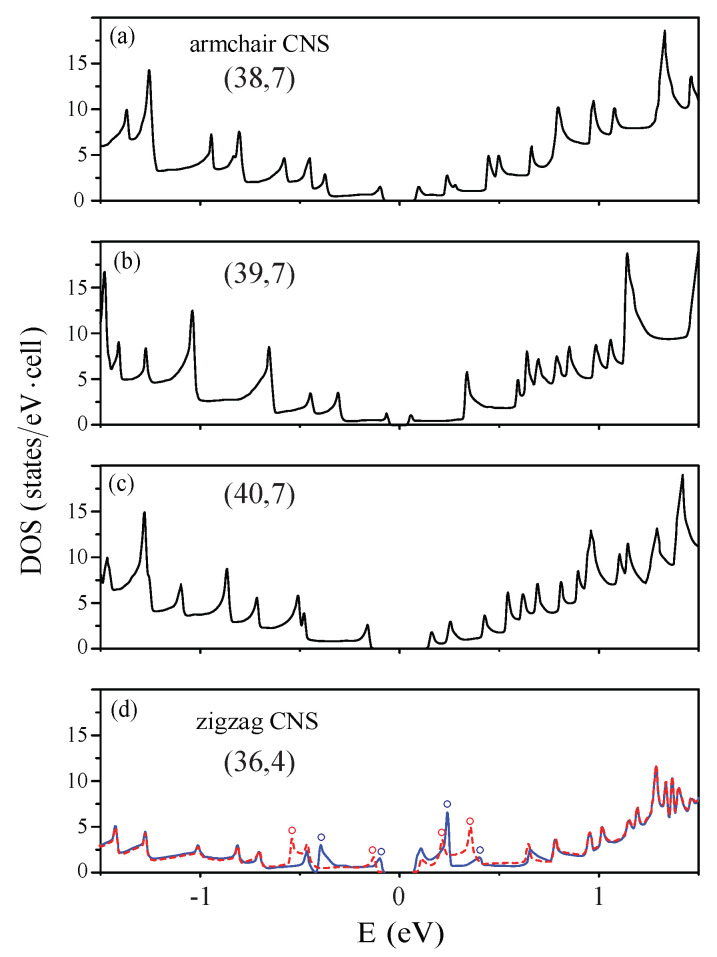
Density of states for the (**a**) (38,7), (**b**) (39,7) and (**c**) (40,7) armchair nanoscrolls, and (**d**) for the (36,4) zigzag one.

The main features of DOSs in carbon nanoscrolls are mainly determined by the complex cooperation relation among the edge structure, total width, and internal length, as clearly shown in Figure 7a–d.

The van Hove singularities only come from the parabolic energy dispersions (band structures in Figure 4a–c), leading to the square-root pronounced peaks. The valence and conduction peaks closest to the Fermi level form an energy gap corresponding to a semiconducting nanoscroll system. The asymmetric peak structures about E=0 are very apparent; furthermore, a simple relation in energy spacing of two neighboring prominent peaks is absent. That is to say, it is very difficult to identify a specific one-to-one correspondence in peak and geometric structures. Both HRTEM and STS need to be utilized to examine the theoretical predictions on the geometric and electronic properties. There are no spin-split peaks in armchair nanoscrolls (Figure 7a–c), while they are present in zigzag systems (blue and red circles in Figure 7d). The energy splittings, which are due to the partial flat bands at the zone boundary (Figure 7b), are relatively obvious. The SP-STS examinations on them could provide very useful information on the ferromagnetic configurations of zigzag nanoscrolls, being in sharp contrast with degenerate behavior from the anti-ferromagnetic ones of pristine zigzag graphene nanoribbons.

### 3.5. Comparisons among the Planar, Curved/Zipped, Folded and Scrolled Systems

**Figure 8:**
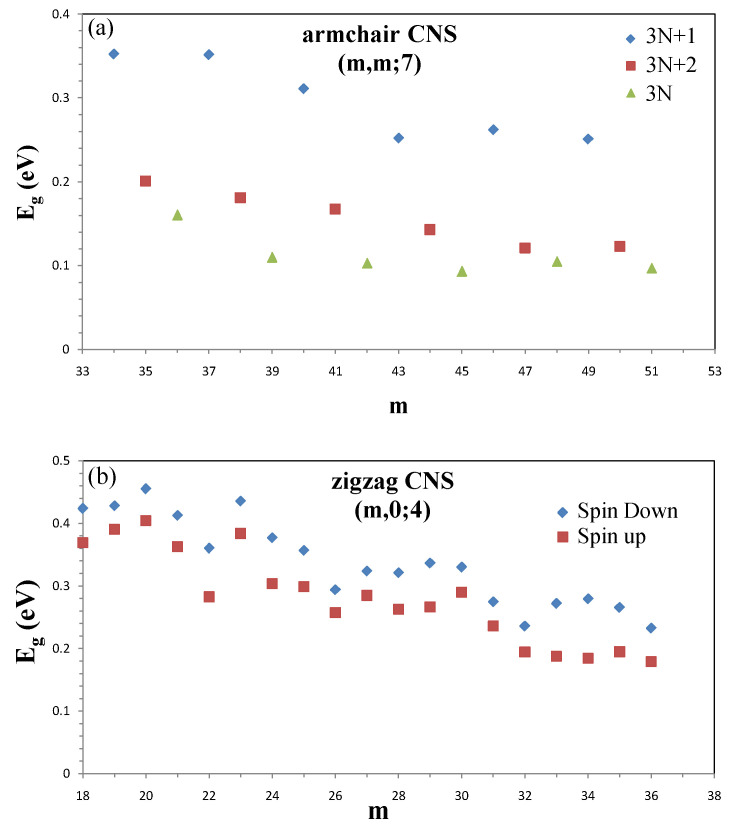
The width-dependent energy gaps for (**a**) armchair carbon nanoscrolls with $N_{in}=7$ and (**b**) zigzag systems with $N_{in}=4$.

The flexible carbon honeycomb lattice can be presented in various forms under a very strong σ bonding. Such structures create the diverse essential properties and thus induce important differences among the planar, curved, folded, and scrolled graphene nanoribbons. For armchair nanoribbons, only parts of the curved systems exhibit the 1D metallic property, mainly owing to the edge–edge interactions [40,41]. Similar behavior is revealed in the even-zAA stacking of the folded zigzag systems [55]. The valence and conduction bands, which determine the metallic or semiconducting properties, are very sensitive to the geometric structure. All the planar and folded armchair systems have the parabolic bands with direct energy gaps at $k_{x}=0$ [55]. However, the curved and scrolled ones might possess non-monotonous energy dispersions with direct or indirect energy gaps (Figure 8b).

Those of zigzag systems belong to the partially flat edge-localized bands at $k_{x}>2/3$. An obvious spin splitting appears near the Fermi level when the magnetic environments are different for spin-up and spin-down states near the open edges, e.g., for folded odd-zAB and scrolled zigzag nanoribbons. Specifically, only the folded even-zAB stacking presents a pair of linearly intersecting energy bands at $k_{x}∼2/3$, as observed in armchair carbon nanotubes.

The width dependences of energy gaps are greatly enriched by the geometric structures. There are three categories in the planar and scrolled armchair nanoribbons (Figure 8a), but six categories in the folded systems. In addition to $N_{A}=3I$, 3I+1, and 3I+2, the last ones also depend on the odd/even number of dimer lines, where $N_{A}$ is the ribbon width and I is an integer. For $N_{A}=3I+2$, the planar systems have the smallest energy gaps because of the finite-size confinement. However, the opposite is true for the scrolled systems under the combined effects. In comparisons among the various pristine systems, the highest energy gaps are revealed in the even-aAA${}^{\prime }$ folded armchair nanoribbons of $N_{A}=6I+4$. As for zigzag nanoribbons, only the scrolled and odd-zAB folded systems present the spin–split energy gaps. The width-dependent declining behavior is obvious except for the folded even-zAB stacking systems with the strong edge–edge interactions. Furthermore, the wave-like fluctuation comes to exist in the scrolled systems.

## 4. Conclusions

After the self-consistent field is solved, the stable structure is determined by the equilibrium between the ribbon width and the scroll surface. If the internal length or the layer–layer overlapping area is too small, the nanoscroll structure will not be sustainable. In consequence, the typical cross-section of the ideal scroll becomes more oval. For armchair CNSs, the minimum (critical) internal length is N${}_{in}=7$. Given an internal length smaller than the critical length, the optimal structure will be restored back to the shape where the internal length is critical. With respect to each internal length, there exists a stable structure with the minimum ribbon width. Assuming that the ribbon width is less than the minimum value, the scroll structure will collapse to the flat graphene nanoribbon due to the insufficient layer–layer interactions. Such interaction will lead to the AA stacking configuration, which provides larger interaction forces, and the average interlayer distance is about 3.35 Å. For a group of CNSs with a small deviation about the curvature radius, all the formations will result in the same internal length in order to maintain the AA stacking configuration. On the other hand, for the zigzag systems, the critical internal length is N${}_{in}=4$, and the optimal configuration is AB stacking with an average interlayer distance of ∼3.2 Å. Such stacking configuration is the same as that in the bilayer graphene and nanoribbon. As compared with armchair CNSs, zigzag CNSs possess larger curvature radii, and layer–layer interactions. Therefore, zigzag CNSs can form with a smaller width ribbon.

The formation energy (ΔE) is mainly dominated by the bending energy and the interlayer interaction. The competition between the bending energy and the interlayer interaction would determine the two critical structure parameters: the internal length and the ribbon width. Note that the E${}_{for}$’s are greater than zero because the bending energy is always larger in magnitude than the interlayer interaction. For the armchair systems, three kinds of N${}_{y}$-dependences of ΔE are found, and the ΔE’s increase with the ribbon width. The relationship is attributed to the increased layer–layer interactions with the larger overlapping areas, yet the decreased bending energies with the larger curvature radius. Hence, the critical ribbon widths of N${}_{in}=7$, 9, and 11 are, respectively, 34, 43, and 47. As for the zigzag system, it presents similarities to the armchair type regarding the energy dependence. However, there are some differences since they are not rolled in the same manner. The zigzag systems possess larger curvatures and stronger layer–layer interactions owing to the AB stacking configuration. As the width increases, the energy decays faster in the AB stacked zigzag system than in the AA stacked armchair system so that the formation energy can easily and quickly reach the deeper negative levels. In conclusion, the lower total energy makes CNS more stable than the flat nanoribbon, and the critical ribbon widths of N${}_{in}=4$ and 9 are, respectively, 18 and 20.

## Figures and Tables

**Table 1:** The energy gaps, wave vectors of highest occupied state (HOS) and lowest unoccupied state (LUS), and the 3N type of various systems.

Systems	Type	$K_{x}$ of HOS/LUS	Energy Gaps
Armchair CNS (38,38;7)	3N+2	0.10/0.10	direct; 0.181 eV
Armchair CNS (39,39;7)	3N	0.01/0.13	indirect; 0.112 eV
Armchair CNS (40,40;7)	3N+1	0.10/0.10	direct; 0.323 eV
Zigzag CNS (36,0;4)	N/A	0.67/0.67	direct; 0.180/0.230 eV

## Data Availability

The data presented in this study are available on request from the corresponding author.
